# Clinical and Genetic Characteristics of Thymoma Patients With Autoimmune Hepatitis and Myocarditis

**DOI:** 10.3389/fonc.2021.746304

**Published:** 2022-01-07

**Authors:** Xin-tao Yu, Lei Yu, Xin Du, Zhen Yu, Xing-guo Yang, Yu-xuan Jiang

**Affiliations:** Department of Thoracic Surgery, Beijing Tongren Hospital, Capital Medical University, Beijing, China

**Keywords:** autoimmune hepatitis, myocarditis, mdm4, p53, thymoma

## Abstract

**Background:**

Our study investigated a special series of thymoma with autoimmune hepatitis and myocarditis and tried to reveal the gene expression profiles of this series of thymoma.

**Methods:**

From 2011 to 2019, a total of 13 special thymoma patients presented with autoimmune hepatitis and myocarditis, accounting for about 1.26% of thymoma patients undergoing surgery in Beijing TongRen Hospital. Clinical data were retrospectively collected. All samples were harvested during surgical procedures, and analyzed to identify changes in gene expression using the CapitalBio mRNA microarray analysis, the Whole exome sequencing analysis (WES), qPCR and immunohistochemistry (IHC) tools.

**Results:**

After surgery, patient symptoms were relieved gradually. Levels of lactate dehydrogenase (LDH), creatine kinase MB (CK-MB), aspartate transaminase (AST), and alanine amiotransferase (ALT) increased to some extent within 1 to 3 months after surgery, and fluctuated, and then, gradually decreased close to normal within 6 months after surgery. Enrichment analysis of Kyoto Genome and Genome Encyclopedia (KEGG) pathway was performed and enrichment results were visualized. It indicated that gene expression of 5 signaling pathways, including cell cycle and p53 signaling pathway, were generally abnormal. P53 expression was up-regulated in all tumor tissues. However, IHC and qPCR analysis showed that there was no significant difference in p21 expression between normal and tumor tissue. Results of WES showed that only one driver gene-*MDM4* amplified 4 fold in 53.2% thymoma cells. Further qPCR and IHC analysis confirmed the up-regulation of the expression of p53 and mdm4 in 13 thymoma patients with autoimmune hepatitis and myocarditis.

**Conclusion:**

Our study reveals the clinical and genetic characteristics of thymoma patients with autoimmune hepatitis and myocarditis. For this special category of thymoma, the up-regulation of p53 and mdm4 plays an important role in the occurrence of thymoma and autoimmune hepatitis/myocarditis.

## Introduction

Thymoma is the most common type of neoplasms of the anterior mediastinum. It’s often associated with a variety of autoimmune diseases (1). The most common autoimmune diseases is the myasthenia gravis (MG), which is characterized by weakness and fatigability of skeletal and extraocular muscles (2). Other thymoma-related autoimmune diseases include Good’s syndrome, dermatomyositis, Addison’s disease, rheumatoid arthritis, and so on (3–6). Autoimmune hepatitis and myocarditis are uncommon presentations of thymoma. The association between thymoma and autoimmune hepatitis/myocarditis has been found in very few cases. Extended thymectomy is an effective way to treat these patients (7). However, the mechanism underlying the development of autoimmune hepatitis/myocarditis in patients with thymoma is unclear. Here, we report a special series of 13 thymoma patients with autoimmune hepatitis and myocarditis, and investigate their clinical and genetic characteristics.

## Materials and Methods

Clinical data were retrospectively collected from the department of thoracic surgery of Beijing TongRen Hospital. From 2011 to 2019, there were 1030 thymoma patients who underwent surgery at Beijing TongRen Hospital. Among these patients, a total of 13 special thymoma patients presented with autoimmune hepatitis and myocarditis, accounting for about 1.26% of thymoma patients undergoing surgery in our department. The diagnostic criteria was according to NCCN Clinical Practice Guidelines in Oncology, American Heart Association and American Association for the Study of Liver Diseases. Diagnostic criteria remained the same in this study. Among the 13 patients, 8 (61.5%) were male and 5 (38.5%) females with an overall median age of 47 years (range 22-72). The patients underwent thymoma resection. Primary thymomas were classified according to the World Health Organization (WHO) criteria as follows: type AB, B1, B2, and B3 (8). The modified Masaoka’s classification was used to stage for these primary thymic tumors.

Laboratory test parameters, such as routine biochemistry and lymphocyte count, were obtained from the Clinical laboratory of Beijing Tongren Hospital. All thymoma samples were harvested during surgical procedures from Beijing Tongren Hospital. Normal thymus tissue and adjacent tumor tissue were used as the control groups. The specimens were immediately frozen in liquid nitrogen and stored at −80°C until use. The protocol for sample collection was approved by the Institutional Review Board of Beijing Tongren Hospital, China. Informed written consent was obtained from each patient before sample collection. All study procedures were conducted in line with the approved protocol.

### Sample Collection and Determination of Lymphocyte Subsets

Fasting venous blood (1-2ml) was collected from thymoma patients before and after operation. 50μl heparin and 20μl of six color fluorescent monoclonal antibodies were added to the blood and mixed at room temperature in the dark for 15 minutes. 450μl hemolysin was added to each tube and BD FACS Lysing was used to dilute it. Samples were mixed at room temperature for 10 minutes and then analyzed by flow cytometry and lymphocyte subsets analysis software. T lymphocyte surface markers were CD3+, CD4+, CD8+, and NK cells markers were CD16+ and CD56+.

### RNA Extraction, Labeling, and Hybridization

Total RNA, containing small RNA, was extracted from thymoma and paraneoplastic thymic carcinoma tissues using the Trizol reagent (Invitrogen) and purified using mirVana miRNA Isolation Kit (Ambion, Austin, TX, USA) following the manufacturer’s instructions. Next, the purity and integrity of RNA were determined using gel electrophoresis whereas RNA concentration was determined spectrophotometrically. Only total RNA samples with RNA integrity number (RIN) values >6 was selected for subsequent analysis.

### Microarray Imaging and Data Analysis

Data from mRNA array was used to analyze data summarization, normalization, and quality control using the GeneSpring software V13.0 (Agilent). Differentially expressed genes were selected if the change of threshold values was ≥2 or ≤−2-folds and if the Benjamini-Hochberg corrected p-values were 0.05.

### KEGG Analysis

The differentially expressed genes obtained from the above analyzes were imported into DAVID 6.8 database (https://david.ncifcrf.gov/) to further examine the differentially expressed pathways. By entering the name list of target genes and selecting species as “Homo Sapiens”, the official names of all target genes were identified. Enrichment analysis of Kyoto Genome and Genome Encyclopedia (KEGG) pathway was performed and enrichment results were visualized.

### RNA Isolation, cDNA Synthesis, and Quantitative PCR Analysis

Tissue samples were homogenized in 1 ml of TRIzol reagent (Invitrogen) using a PowerGen tissue homogenizer. Then, total RNA was isolated from all samples according to the manufacturer’s recommendations. Subsequently, complementary DNA (cDNA) was synthesized from 1µg total RNA using the MMLV Reverse Transcriptase cDNA kit (TAKARA) following the manufacturer’s instructions. Next, quantitative PCR (qPCR) was performed in a 96-well reaction plate using SYBR premix Ex Taq (TAKARA) on a Bio-RadCFX96 real-time PCR detection system (Bio-Rad). The comparative Ct (ΔΔCt) method was used to analyze relative mRNA expression.

### Histologic and Immunohistochemical (IHC) Testing

Immunohistochemical (IHC) staining was performed on 4-μm-thick slides with anti-p53 (ab26, Abcam), anti-p21 (ab109520, Abcam) and anti-mdm4 (ab49993, Abcam) using an automated immunostainer (BenchMark XT, Ventana Medical Systems, Tucson, AZ, USA) following the manufacturer’s protocol. The p53, p21 and mdm4 IHC were interpreted in three tiers: strong nuclear staining in more than 10% of the tumor cells was considered strong positive staining, samples without any nuclear staining of tumor cells (complete absence) were interpreted as negative staining, and cases exhibiting weak, scattered, or patchy positivity were regarded as weak positive staining. Representative images for each category are shown in **Figures 6**, **8**.

### Statistical Analysis

The expression level of p53 in tumor tissues, normal tissues, and tissues adjacent to the tumor was determine using the SPSS 23.0 (IBM, Armonk, NY, USA) and statistical analyses were conducted using Graphpad prism 8 software (Graphpad Software Inc., San Diego, CA, USA). Significance level and misjudgment rate of each KEGG term were estimated by Fisher’s exact and chi-squared (χ2) tests. Measurement data with normal distribution were expressed as mean ± standard deviation, and groups were compared using *t-*tests. For measurement data that did not conform to the normal distribution, rank sum test was used for comparison between groups. Differences were considered statistically significant at a *p*-value of <0.05.

## Results

### Abnormal Symptoms and Auxiliary Examination Results Were Observed in All Patients

Clinical and demographic data of the study population are summarized in **Table 1**. The common clinical characteristic identified in these thymoma patients was that all of them had autoimmune hepatitis and myocarditis. Some patients also presented with other autoimmune diseases, such as autoimmune enteritis, dermatomyositis, Graves’ disease and rheumatoid arthritis. The patients experienced symptoms, such as diarrhea, shortness of breath and fatigue without any known precipitating factors.

**Table 1:** Patients’ information.

Autoimmune disease	Radiotherapy or Chemotherapy	Symptom	Antibody (+)	WHO histology	Masaoka stage	TNM stage	Genetic mutation	Invasion of adjacent mediastinal structures	Autoimmune disease occurs after thymoma
	(Y/N)							(Y/N)	(Y/N)
Autoimmune hepatitis	N	dyspnea fatigue	ANA	B1	II	I	p53	N	Y
Myocarditis			AMA				mdm4		
Autoimmune hepatitis	N	dyspnea	ANA	B2	II	I	p53	Y	Y
Myocarditis		fatigue	AMA				mdm4	(lung)	
Autoimmune enteritis		tenesmus	GAB						
			Ro52						
Autoimmune hepatitis	N	fatigue	ANA	B2	II	I	p53	Y	Y
Myocarditis		dizzy	AMA				mdm4	(chest wall)	
Autoimmune hepatitis	N	nausea	ANA	B1	II	I	p53	N	Y
Myocarditis		jaundice	AMA				mdm4		
Autoimmune hepatitis	N	dyspnea	ANA	AB	I	I	p53	N	Y
Myocarditis		fatigue	AMA				mdm4		
Dermatomyositis		erythra							
Autoimmune hepatitis	Y	dyspnea	ANA	B3	I	I	p53	Y	Y
Myocarditis		fatigue	AMA				mdm4	(pericardium)	
Autoimmune hepatitis	Y	fatigue	ANA	B1	II	I	p53	N	Y
Myocarditis		arthrodynia	AMA				mdm4		
Rheumatoid arthritis		fever	SSA						
			CCP						
Autoimmune hepatitis	N	dyspnea fatigue	ANA	B2	I	I	p53	N	Y
Myocarditis			AMA				mdm4		
Autoimmune hepatitis	Y	dyspnea fatigue	ANA	B1	II	I	p53	Y	Y
Myocarditis			AMA				mdm4	(phrenic nerve)	
Autoimmune hepatitis	Y	dyspnea fatigue	ANA	B2	II	I	p53	Y	Y
Myocarditis		chromatosis	AMA				mdm4	(left innominate vein)	
Graves’ disease		hypotension	ACA						
Autoimmune hepatitis	N	dyspnea fatigue	ANA	AB	II	I	p53	N	Y
Myocarditis			AMA				mdm4		
Autoimmune hepatitis	N	dyspnea fatigue	ANA	B3	III	II	p53	Y	Y
Myocarditis			AMA				mdm4	(lung)	
Autoimmune hepatitis	N	dyspnea fatigue	ANA	B3	III	II	p53	Y	Y
Myocarditis		diminution of vision	AMA				mdm4	(pericardium)	
			SSA						
			Ro52						
			Jo-1						

ANA, anti-nuclear antibody; AMA, anti-mitochondrial antibody; ACA, anti-cardiolipin antibody; CCP, cyclic citrullinated peptide.

Ultrasonic cardiogram revealed that most patients had an ejection fraction (EF) of 70%, with a slightly enlarged left atrium, aortic sinus expansion, and aortic valve regurgitation. However, the echocardiography results of several patients demonstrated a diffuse myocardial wall hypokinesis with reduced left ventricular ejection fraction (LVEF) of less than 35%.

Laboratory examination showed that all patients who received preoperative examination had multiple autoantibody-positive statuses for antinuclear antibody (ANA), anticardiolipin antibody (ACA), antimitochondrial antibody (AMA), Ro52, etc. Their serological tests showed abnormally elevated levels of myocardial enzymes like LDH, AST, ALT, and CK-MB before surgery. Immunophenotyping tests on lymphocyte subsets identified some abnormal cells, such as CD3+CD4+ helper T cells (TH), CD3+CD8+ suppressor T cells (TS).

### Extended Resection of Thymoma Relieved Symptoms of Autoimmune Diseases and Reduced Hepatic and Cardiac Injury

**Figure 1:**
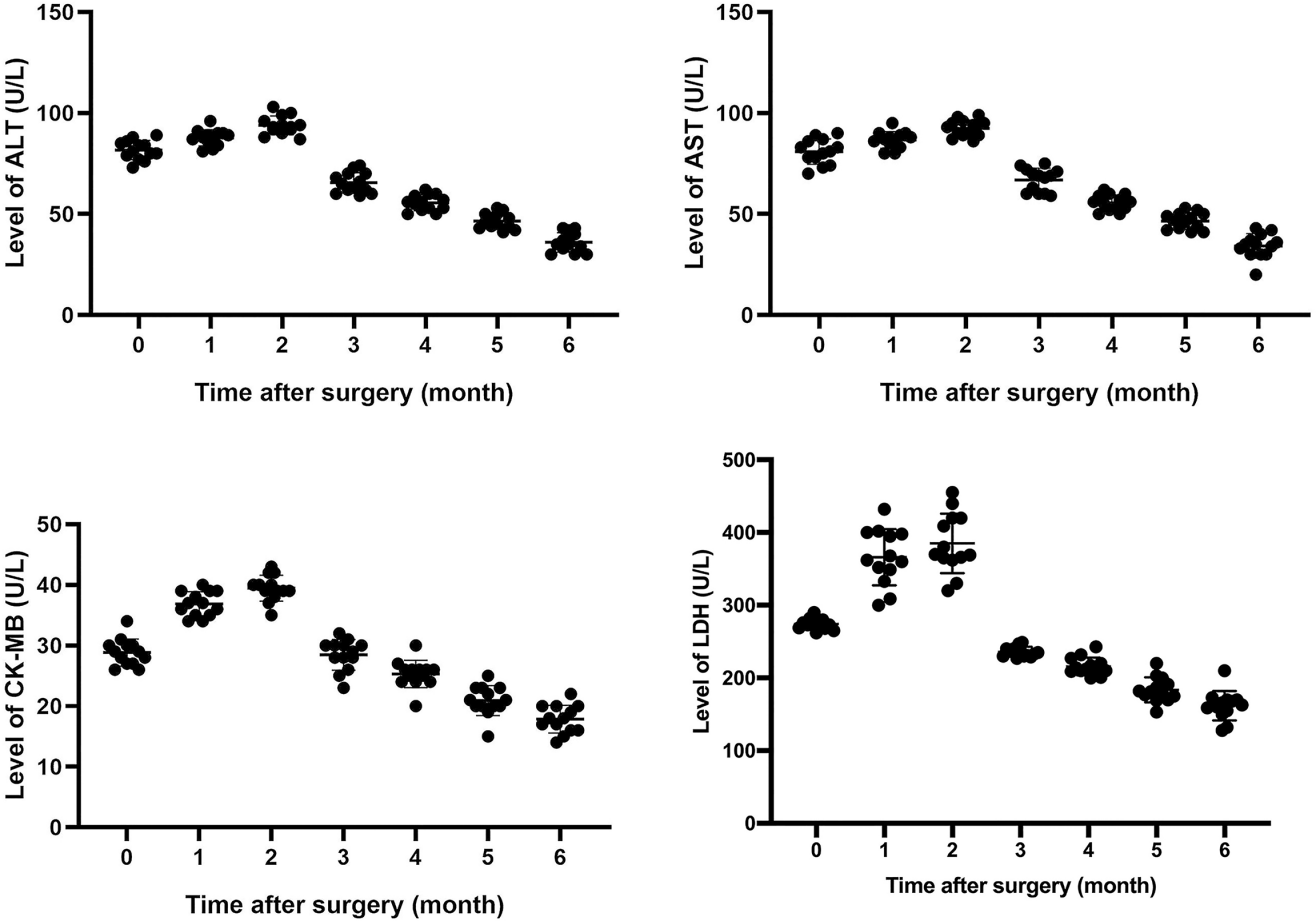
Changes of different enzymes’ levels after surgery.

All patients underwent extended resection of thymoma. After surgery, abnormal symptoms disappeared gradually. It was observed that levels of LDH, CK-MB, AST, and ALT increased to some extent within 1 to 3 months after surgery, and fluctuated, and then, gradually decreased close to normal within 6 months after surgery (**Figure 1**). As shown in **Table 2**, the same trend was observed in the percentages of total CD3+ T cells and CD4+/CD8+ T cells ratio.

**Table 2:** T lymphocyte subsets analysis of 13 patients ( $\bar{\text{x}}$ ± *s*).

	preoperative%	postoperative% (6 months)	*t* value	*p* value
Total T-lymphocytes (CD3+)	91.92 ± 5.30	80.23 ± 2.56	8.77	<0.01
TH (CD3+CD8+	51.10 ± 1.24	33.6 ± 2.32	28.27	<0.01
TS (CD3+CD4+	28.20 ± 3.87	59.01 ± 12.25	-7.34	<0.01
CD4+/CD8+T-lymphocytes ratio	0.55 ± 0.08	1.75 ± 0.34	-10.96	<0.01
Treg (CD4+CD25+foxp3+)	6.98 ± 1.31	6.85 ± 1.36	0.23	0.822
NK (CD3-CD16+56+	9.76 ± 2.95	8.81 ± 2.04	1.86	0.088

### mRNA Profiles Differing Between Thymoma and Controls

**Figure 2:**
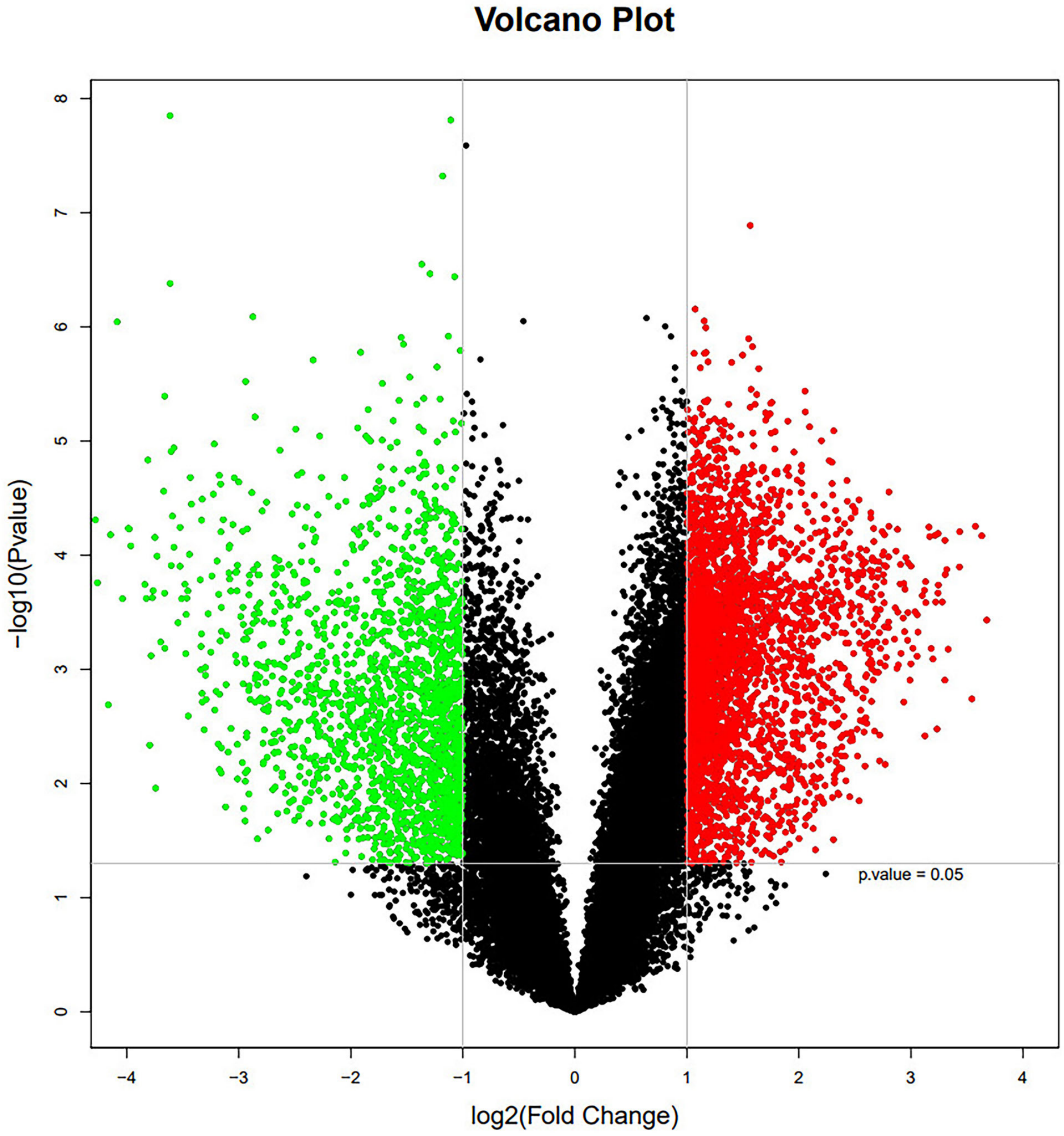
The mRNA expression profile in thymoma patients and normal thymic tissue controls. Red and green dots stand for up-regulated and down-regulated genes, respectively.

To further investigate molecular mechanisms, mRNA samples from 13 thymoma patients and 13 normal thymus tissue controls were analyzed using the Agilent Whole Human Genome Microarray. A scatter plot revealed notable differences between the two groups as illustrated in **Figure 2**.

### Disease Analysis

**Figure 3:**
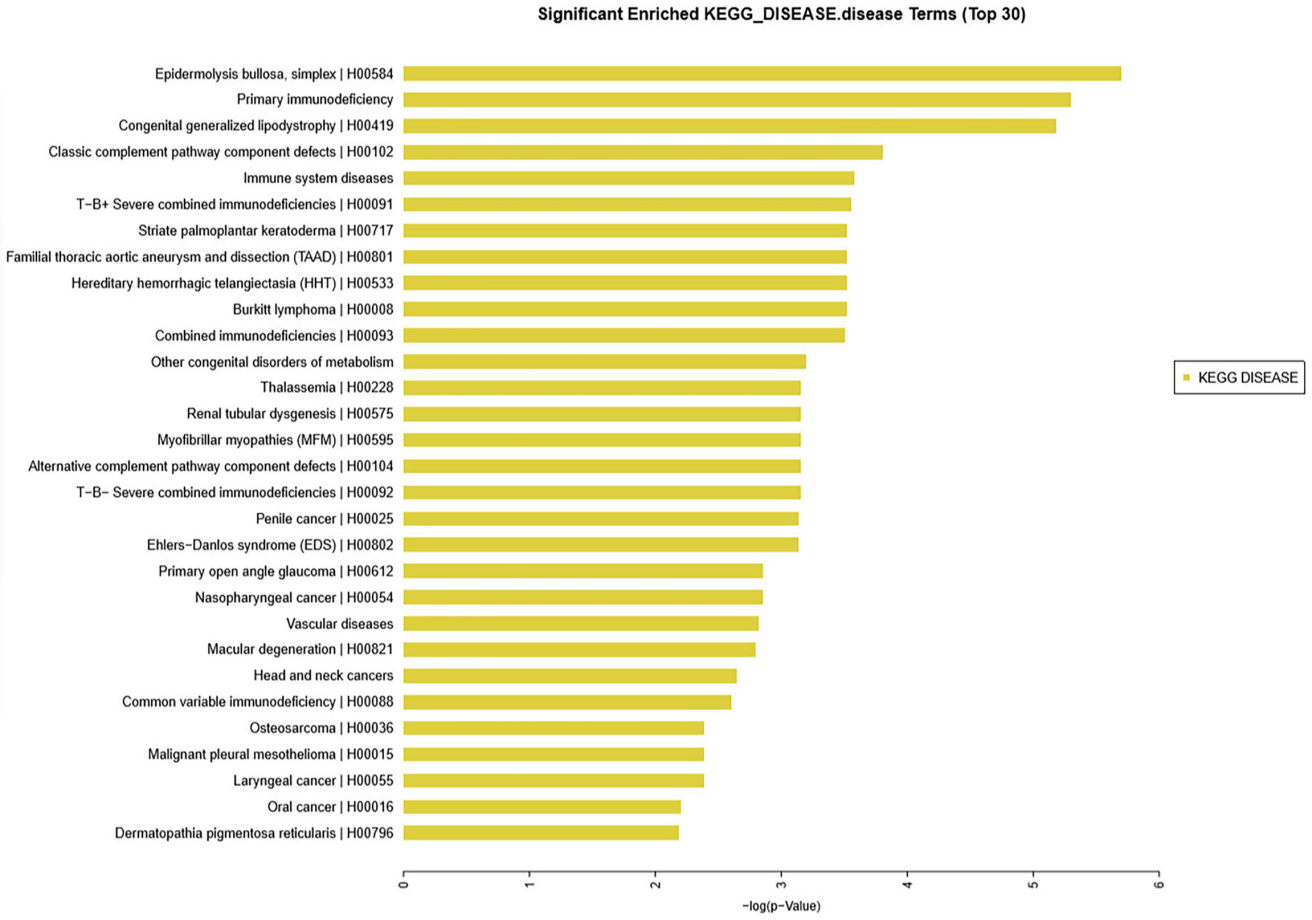
KEGG database analysis identified the top 30 diseases in 13 patients with thymoma unlike control patients. Among such diseases were epidermolysis bullosa, primary immunodeficiency, congenital generalized lipodystrophy, classic complement pathway component defects, and immune system diseases.

KEGG analysis was conducted for the significantly differential expression genes. Significantly altered mRNAs were analyzed from our microarray datasets. Results showed that the top 30 diseases included immune system diseases (**Figure 3**), which is consistent with the basic information of the 13 patients.

### Several Pathways Had a Close Relation With the 13 Patients

**Figure 4:**
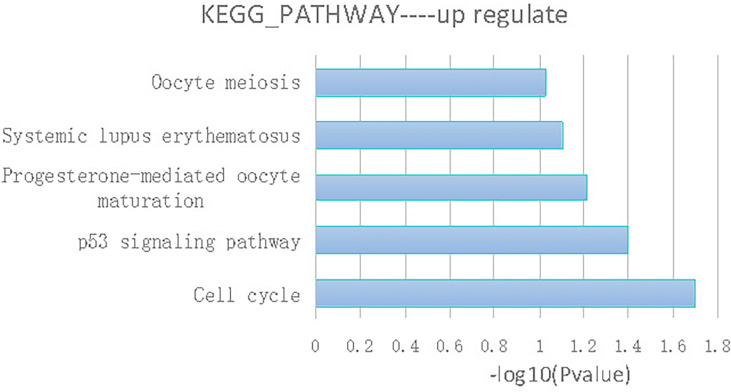
KEGG database analysis indicated that gene expression of 5 signaling pathways including cell cycle, p53 signaling pathway, progesterone mediated oocyte maturation, systemic lupus erythematosus and oocyte meiosis were generally abnormal.

Pathway’s analysis revealed top 5 abnormal signaling pathways in thymoma tissues. Among the pathways, cell cycle pathway has been shown to play a vital role in the formation of thymoma tumor cells (**Figure 4**).

### P53 and p21 Gene Which Mainly Regulate Cell Cycle Pathway Were Tested in Thymoma Tumor Cells

The expression of p53 and p21 in thymoma tissues of 13 patients was determined using qPCR and IHC assays.

**Figure 5:**
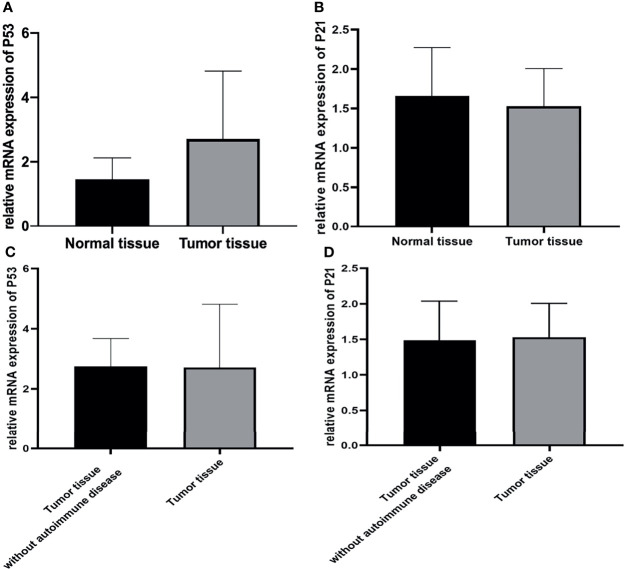
**(A, C)** The expression of p53 was higher in all tumor tissues than in normal tissues. (p<0.05), but it was similar between tumor tissue with autoimmune hepatitis and myocarditis and tumor tissue without autoimmune disease. (p>0.05) **(B, D)** The expression of p21 was similar between normal and all tumor tissues. (p>0.05).

Firstly, the expression of p53 and p21 at the RNA level was explored using real-time qPCR. The results indicated that p53 expression was up-regulated in all tumor tissues from the patients. However, there was no significant difference in p21 expression between normal and all tumor tissues (**Figure 5**).

**Figure 6:**
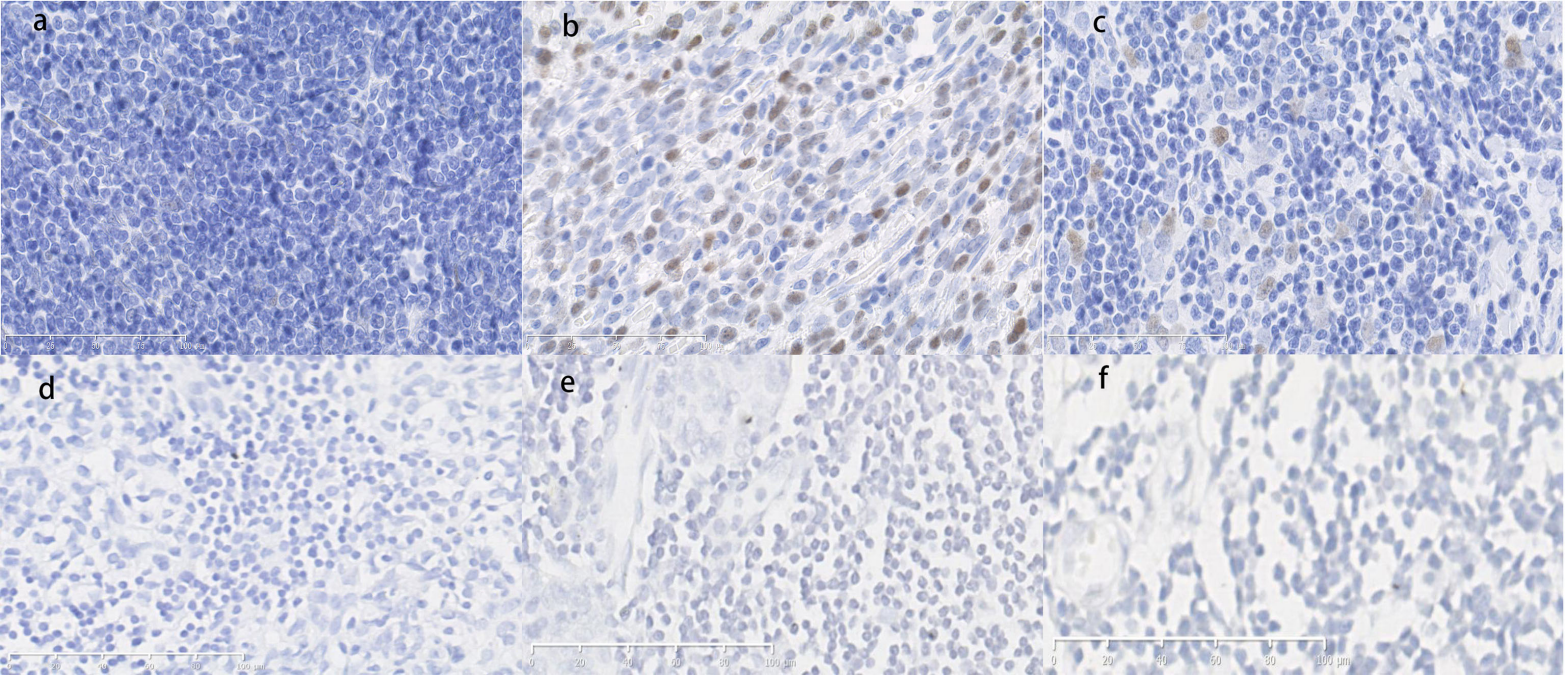
**(A)** Normal thymus tissue immunostained with p53 antibodies. Score=0, low expression. **(B)** Thymoma tissue with hepatitis/myocarditis immunostained with p53 antibodies. The p53-positive cells are presented in the field. Score=9, high expression. **(C)** Tumor tissue without autoimmune disease immunostained with p53 antibodies. Score=2, low expression. **(D)** Normal thymus tissue immunostained with p21 antibodies. Score=0, low expression. **(E)** Thymoma tissue with hepatitis/myocarditis immunostained with p21 antibodies. Score=0, low expression. **(F)** Tumor tissues without autoimmune disease immunostained with p21 antibodies. Score=0, low expression. (×400).

P53-positive cells were observed in the frozen sections of the tumor tissues with autoimmune hepatitis/myocarditis and the tumor tissues without autoimmune disease based on immunohistochemistry. As shown in **Figure 6**, p53-positive cell was not detected in some epithelial-like cells located in the medulla for normal thymus tissues, whereas for thymoma tissues, p53-positive cells were detected in medulla regions. On the contrary, there weren’t p21-positive cells in both normal and tumor tissues (**Figure 6**).

### Mdm4 Expression Was Up-Regulated in Tumor Tissue With Autoimmune Hepatitis and Myocarditis

**Figure 7:**
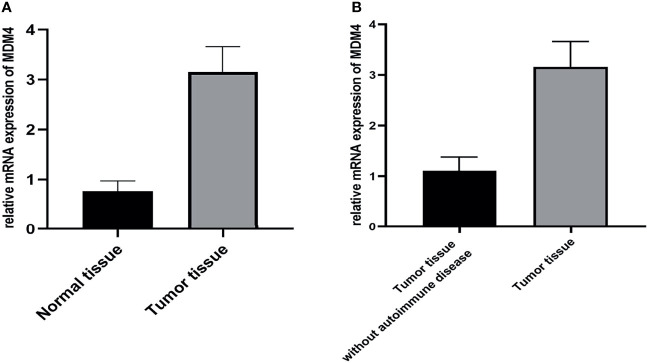
**(A, B)** Results of qPCR showed that mdm4 was up-regulated in tumor tissues comparing with normal tissue and tumor tissue without autoimmune disease. (p<0.05).

The expression of mdm4 was explored at the RNA level using qPCR. The result indicated that the level of mdm4 was up-regulated in tumor tissues with autoimmune hepatitis and myocarditis comparing to the normal thymus tissue and tumor tissues without autoimmune disease (**Figure 7**).

**Figure 8:**
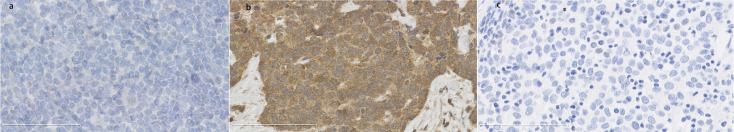
**(A)** Normal thymus tissue immunostained with mdm4 antibodies. Score=0, low expression. **(B)** Thymoma tissue with hepatitis/myocarditis immunostained with mdm4 antibodies. Mdm4-positive cells were shown in the field. Score=12, high expression. **(C)** Tumor tissues without autoimmune disease immunostained with p21 antibodies. Score=0, low expression. (×400).

Using IHC, mdm4 staining was negative in epithelial-like cells located in the medulla for the normal thymus tissue and tumor tissues without autoimmune disease. In contrast, more mdm4-positive cells were found in medulla regions for tumor tissues with autoimmune hepatitis and myocarditis (**Figure 8**).

## Discussion

Thymoma, a mediastinal malignant tumor, has often been associated with autoimmune diseases. Previous studies have shown that many autoimmune diseases, such as Hashimoto’s thyroiditis, Isaac’s syndrome, and Morvan syndrome were caused by thymoma (9). Sometimes, surgical treatment might result in good prognosis of thymoma combined with non-myasthenia gravis autoimmune diseases (10–13). Here, we report the clinical and genetic characteristics of 13 special thymoma patients with autoimmune hepatitis and myocarditis.

Before surgery, these 13 patients had abnormal assay index of LDH, CK-MB, AST, ALT and lymphocyte subsets, which might be attributed to thymoma, autoimmune hepatitis and myocarditis. We continued to trace on the change of these enzymes’ levels for 6 months after extended resection for thymoma. The follow-up results indicated that levels of LDH, CK-MB, AST, and ALT rose to some extent within 1 to 3 months after surgery, and then, gradually decreased close to normal. It provided evidence that there exists a link between thymoma and autoimmune hepatitis/myocarditis.

To interpret the results at the genetic level, we conducted microarray analysis to examine differential gene expression profiles of human mRNAs in patients with thymoma and autoimmune hepatitis/myocarditis. And then, we took the differential genes a step further.

As a transcriptional factor, p53 is activated in response to cell stress and regulates many genes involved in cell cycle control, apoptosis, angiogenesis, and cell senescence (14). P53 was reported to be one of the most significantly mutated gene in thymic carcinoma and thymoma (15). Our study revealed that p53 was highly expressed in thymoma patients with autoimmune hepatitis/myocarditis. Results of KEGG database analysis also indicated that cell cycle, p53 signaling pathway and so on were abnormal, similar to our previous study.

Next step, we selected the downstream gene of p53 pathway, p21, to try further revealing the mechanism between thymoma and autoimmune hepatitis/myocarditis. The p21 gene is located on the short arm of chromosome 6 downstream of the p53 pathway. Usually, an abnormal expression of its protein affects the regulation of Cyclin, CDK, and kinase activity, thus affecting cell proliferation and differentiation (16). Previously, some researchers proved that this gene was up-regulated in dermatomyositis (17). But in our study, there was no obvious difference of p21’s expression between thymoma and normal tissue.

Meanwhile, the whole exome sequencing analysis (WES) result indicated that the MDM4 amplifified in this thymoma cells (18). Mdm2 and mdm4 proteins form heterodimers that are much more effective in regulating p53 (19). We ever tested that mdm4/mdm2 heterodimers were down-regulated in thymoma patients without autoimmune hepatitis/myocarditis. However, we used the qPCR assay and IHC test to measure mdm4 levels in these 13 thymoma patients with autoimmune hepatitis/myocarditis, and discovered that mdm4 level was up-regulated. Jonathan P. McNallya’s research group found that potentiation of p53 (via inhibition of mdm2) led to the selective elimination of activated and pathological T cells *in vivo*, which had an impact on the immune system (20). It has also been reported that the *mdm4/mdm2* heterodimers are upregulated not only in the brain and nerve tumor tissues, breast cancer, soft tissue sarcomas, type 1 diabetes, and systemic lupus erythematosus (21–23), but also in the parathyroid glands of patients with renal secondary hyperparathyroidism (24). Therefore, we think that there was a strong association between the up-regulation of p53 and mdm4 and thymoma, as well as autoimmune hepatitis/myocarditis. We can also infer that abnormal p53-mdm4 pathway may promote the occurrence and development of thymoma, which further cause autoimmune hepatitis and myocarditis.

## Conclusion

Our study reveals the clinical and genetic characteristics of thymoma patients with autoimmune hepatitis and myocarditis. For this special category of thymoma, the up-regulation of p53 and mdm4 plays an important role in the occurrence of thymoma and autoimmune hepatitis/myocarditis.

## Data Availability Statement

The datasets presented in this study can be found in online repositories. The names of the repository/repositories and accession number(s) can be found in the article/supplementary material.

## Ethics Statement

Because all patients in this study signed consent forms and were enrolled, informed consent was obtained from all participants. The study was approved by the Human Research Ethics Board of Beijing Tongren Hospital, Capital Medical University, and all experiments were performed in accordance with relevant guidelines and regulations.

## Author Contributions

X-tY and LY: Conceptualization, Methodology, Software. X-tY: Data curation, Writing- Original draft preparation. XD: Visualization, Investigation. ZY: Supervision. X-gY: Software, Validation. Y-xJ: Writing- Reviewing and Editing. All authors contributed to the article and approved the submitted version.

## Funding

This study was supported by the Clinical Technology Innovation Project of Beijing Hospital Authority (XMLX201839) and the National Natural Science Foundation of China (Grant Number: 81550004).

## Conflict of Interest

The authors declare that the research was conducted in the absence of any commercial or financial relationships that could be construed as a potential conflict of interest.

## Publisher’s Note

All claims expressed in this article are solely those of the authors and do not necessarily represent those of their affiliated organizations, or those of the publisher, the editors and the reviewers. Any product that may be evaluated in this article, or claim that may be made by its manufacturer, is not guaranteed or endorsed by the publisher.
